# Role of Seminal microRNAs Detected by Quantitative Polymerase Chain Reaction (qPCR) in Male Infertility: A Systematic Literature Review

**DOI:** 10.7759/cureus.96118

**Published:** 2025-11-05

**Authors:** Manoharan Shunmuga Sundaram, Sanjeeva Reddy, Vettriselvi Venkatesan, Madan Kalagara, Radha Ganesh, Manjula G

**Affiliations:** 1 Reproductive Medicine and Surgery, Sri Ramachandra Institute of Higher Education and Research, Chennai, IND; 2 Human Genetics, Sri Ramachandra Institute of Higher Education and Research, Chennai, IND; 3 Pathology, Vijaya Medical Centre, vishakapatnam, IND

**Keywords:** expression, infertility, microrna, quantitative pcr, seminal plasma, spermatozoa

## Abstract

Male infertility is one of the major causes of conception issues globally. Traditionally, semen analysis serves as the primary diagnostic tool for evaluating male infertility, but it does not provide insights into underlying causes. In recent years, the non-coding short microRNAs (miRNAs) have gained attention because of their stability and regulatory role in sperm development, suggesting strong potential as biomarkers for infertility evaluation. This systematic review seeks to examine the role of seminal miRNAs, quantified through quantitative polymerase chain reaction (qPCR), in relation to male infertility outcomes. Following Preferred Reporting Items for Systematic reviews and Meta-Analyses (PRISMA) guidelines, a literature search was conducted across PMC PubMed, Wiley Online Library, ScienceDirect, and Google Scholar for studies published between 2010 and 2025. Articles reporting miRNA expression in spermatozoa and seminal plasma of infertile men using qPCR were included. A total of 28 articles were included, assessing 1,699 infertile men, of whom 1,239 men were categorized by abnormalities such as oligozoospermia, asthenozoospermia, teratozoospermia, oligoasthenozoospermia, asthenoteratozoospermia, oligoasthenoteratozoospermia, and azoospermia. A differential expression pattern of miRNAs was observed in male infertility. Such an expression pattern was positively or negatively correlated with genes and gene products involved in sperm development, production, and maturation, influencing sperm characteristics. Distinct miRNAs are associated with specific male infertility types, supporting their potential role as diagnostic biomarkers for clinical use.

## Introduction and background

Infertility, described as the failure to conceive after a year of consistent and unprotected sexual activity, is a worldwide health issue. It impacts around 10-15% of couples in their reproductive years, with male-related causes accounting for roughly 20-30% of these cases [1]. Sperm cells or spermatozoa are highly specialized cells tasked with transporting the male’s hereditary unit to the egg during the fertilization process. The proper functioning of sperm determines the ability of a male to reproduce [2]. Three critical factors of sperm, namely, physical form (morphology), ability to move (motility), and number (concentration), influence the fate of fertilization [3]. The probabilities of achieving successful fertilization could be lowered, and male fertility could be impaired by deviations in any of these sperm parameters. Infertile men can exhibit various sperm-related abnormalities, such as teratozoospermia (abnormal sperm shape), asthenozoospermia (poor sperm motility), oligozoospermia (low sperm count), azoospermia (no sperm present in the ejaculate), or a combination of these conditions [4].

Several factors, such as intricate molecular signaling pathways, coordinated gene expression, and genetic integrity, regulate the complex and tightly controlled process occurring during spermatogenesis. It is through this process that sperm cells are formed within the testes, which are then matured in the epididymis [5]. Impaired sperm production or primary testicular failure is frequently caused by genetic defects, namely, mutations in genes essential for spermatogenesis or chromosomal anomalies [6]. However, even with progress in diagnostic techniques, a significant percentage of cases (ranging from 6% to 37%) are still classified as unexplained male infertility, characterized by inferior semen quality without an underlying cause [7].

Conventional semen analysis, which assesses the shape (morphology), movement (motility), and count (concentration) of sperm, continues to serve as the primary diagnostic tool for evaluating male infertility [8]. Despite its widespread use, traditional semen analysis has substantial limitations. A substantial overlap in semen characteristics exists between fertile men and infertile men, and fertility is not ensured via normal semen parameters. As a result, growing attention is being directed towards discovering molecular biomarkers, such as RNAs and specific proteins, which could enhance diagnostic precision and facilitate personalized treatment approaches [9].

MicroRNAs (miRNAs) have been found as especially promising candidates in this group of molecular markers. These miRNAs are non-coding, single-stranded, and short RNA molecules, which are typically 18-24 nucleotides long. miRNAs attach to complementary sequences in the 3′ untranslated regions (UTRs) of target messenger RNAs (mRNAs) and subsequently control gene expression after transcription [10]. This binding leads to either the breakdown of mRNA or the inhibition of its translation, affecting a wide range of cellular activities, such as embryonic development, differentiation, programmed cell death, and cell growth [11]. Spermatogenesis comprises essential phases ranging from the proliferation of spermatogonia and meiotic division to the differentiation of spermatids and the maturation of sperm. These phases are regulated by various miRNAs, such as miR-221, miR-203, and miR-34b-5p, in the male reproductive system [12]. In addition to their role in spermatogenesis, miRNAs such as miR-150, miR-133b, miR-541-5p, and let-7 are involved in regulating the ability to fertilize an egg, capacitation, and sperm motility development. Disruptions in the production or expression of miRNAs have been constantly associated with male infertility and reduced sperm function [13-15].

Differential expression of specific miRNAs has been observed between infertile and fertile men, highlighting their regulatory role in semen quality. For example, miR-21 and miR-22 have been shown to negatively correlate with seminal pH and normal sperm morphology [15], while miR-23a-3p and miR-23b-3p were inversely associated with sperm morphology in men diagnosed with oligoasthenozoospermia [16]. By contrast, miR-34c-5p, miR-181a, and miR-122 have shown positive correlations with sperm morphology and concentration in patients diagnosed with oligoasthenoteratozoospermia [17]. These results underscore the intricate nature of miRNA-mediated regulation, indicating that some miRNAs may enhance sperm quality, while others may impair it, depending on the specific type of infertility. 

Profiling miRNA expression may enhance diagnostic accuracy beyond standard semen analysis. This systematic literature review (SLR) aims to (1) evaluate differential miRNA expression between fertile and infertile men to identify potential diagnostic or prognostic biomarkers and (2) investigate miRNA expression in spermatozoa and seminal plasma across infertile conditions, including oligozoospermia, asthenozoospermia, teratozoospermia, and their combinations.

## Review

Methodology

The present systematic literature review was registered with PROSPERO (CRD42021250912) and followed PRISMA guidelines. 

PICO Framework

The PICO framework of this study is as follows: Population (P): adult men diagnosed with infertility (oligospermia, azoospermia, asthenozoospermia, teratozoospermia); Intervention (I): detection of miRNA expression in semen or seminal plasma via quantitative PCR (qPCR); Control (C): fertile men with normal semen parameters; Outcome (O): differential miRNA expression and potential diagnostic/prognostic value. 

Selection Criteria

The inclusion criteria include human male subjects with infertility, spermatozoa or seminal plasma samples, miRNA quantification via qPCR/RT-qPCR, and peer-reviewed full-text articles in English (2010-2025). The exclusion criteria are reviews, editorials, abstracts, book chapters; studies using microarrays or NGS without qPCR validation; non-semen samples; animal or cell line studies; and non-English texts or inaccessible full-texts. 

Search Strategy

A systematic search was conducted across PubMed Central (PMC), ScienceDirect, Wiley Online Library, and Google Scholar to identify relevant articles published between January 2010 and March 2025. The search combined controlled vocabulary (MeSH terms where applicable) and free-text keywords related to microRNAs and male infertility. Boolean operators (“AND,” “OR”) were applied to connect concepts, and filters were used to restrict results to open-access, English-language, peer-reviewed journal articles. 

PubMed Central: The search was performed using both MeSH terms and Title/Abstract fields to capture studies related to microRNA expression in male reproductive samples: (“MicroRNAs”) AND (“Semen” OR “Spermatozoa”) AND (“Infertility, Male” OR “Oligospermia” OR “Asthenozoospermia” OR “Azoospermia”) AND (“Gene Expression Regulation”). To broaden the scope, the following free-text strategy was used: ((((microRNA[Title/Abstract]) OR (microRNAs[Title/Abstract])) OR (miR[Title/Abstract])) OR (miRNA[Title/Abstract])) OR (miRNAs[Title/Abstract]).

This initial search was refined by adding terms related to expression, seminal plasma, spermatozoa, sperm, semen, and infertility-related conditions (oligospermic, asthenozoospermia, azoospermia, asthenoteratozoospermia, normozoospermic). The final PubMed Central search string was (((((((MicroRNAs) OR microRNA) OR miR) AND seminal plasma) OR spermatozoa) OR sperm) OR seminal) OR semen) AND expression) OR expressions) AND oligospermic) OR azoospermia) OR asthenozoospermia) OR normozoospermic) OR asthenoteratozoospermic. Filters applied were open access and 2010-2025. The hits retrieved were 703.

ScienceDirect: Search terms were applied to article titles, abstracts, and keywords: (microRNA OR microRNAs OR miR OR micro-ribonucleic acid OR micro ribonucleic acid) AND (seminal plasma OR spermatozoa OR sperm OR seminal) AND (expression). The search was further refined with infertility-related terms (asthenozoospermia, oligoasthenozoospermia, oligospermic, azoospermia). Filters applied were research articles, English language, open access, and open archive; subject areas of Biochemistry, Genetics and Molecular Biology, Medicine, Dentistry, Immunology, Microbiology, Pharmacology, Toxicology, and Pharmaceutical Sciences; and 2010-2025. Hits retrieved were 711.

Wiley Online Library: The search was carried out using the “Anywhere” field with combinations of microRNA and male infertility-related terms: (microRNA OR microRNAs OR miR OR micro-ribonucleic acid OR micro ribonucleic acid OR miRNA OR miRNAs) AND (seminal plasma OR spermatozoa OR sperm OR seminal) AND (expression) AND (asthenozoospermia OR oligoasthenozoospermia OR oligospermic OR azoospermia OR normozoospermic OR asthenoteratozoospermic). Filters applied were open access journal content and 2010-2025. Hits retrieved were 5.

Google Scholar: A simplified keyword search was conducted using the allintitle operator to identify open-access, non-review studies: (microRNA OR microRNAs OR miR OR micro-ribonucleic acid OR micro ribonucleic acid OR miRNA OR miRNAs) AND (expression) AND (seminal plasma OR spermatozoa OR sperm OR seminal) AND (infertility OR infertile). Filters applied were 2010-2025, excluding review articles. Hits retrieved were 17.

Summary of search terms: The key terms used across databases included “microRNA” OR “miR” AND “seminal plasma” OR “spermatozoa” OR “sperm” OR “seminal expression” AND “asthenozoospermia” OR “oligoasthenozoospermia” OR “azoospermia” AND “infertile” OR “infertility”. Duplicates were removed, and all retrieved citations were screened according to inclusion and exclusion criteria.

Data Extraction

Two reviewers independently extracted data using the JBI (Joanna Briggs Institute) data extraction sheet [18]. From each included study, data were systematically extracted on the following parameters: study characteristics, participant details, sample type, miRNAs investigated, differential expression, associations with sperm parameters, and gene/hormone correlations. Any variance was solved by discussion.

Risk-of-Bias Quality Assessment

Risk of bias (RoB) was assessed using QUADAS-2 as recommended by Cochrane for evaluating the quality of diagnostic accuracy studies [19]. Two reviewers independently assessed each study across four domains: patient selection, index test, reference standard, and flow/timing. Judgments were categorized as low, high, or unclear risk of bias. 

Results

Literature Screening

**Figure 1:**
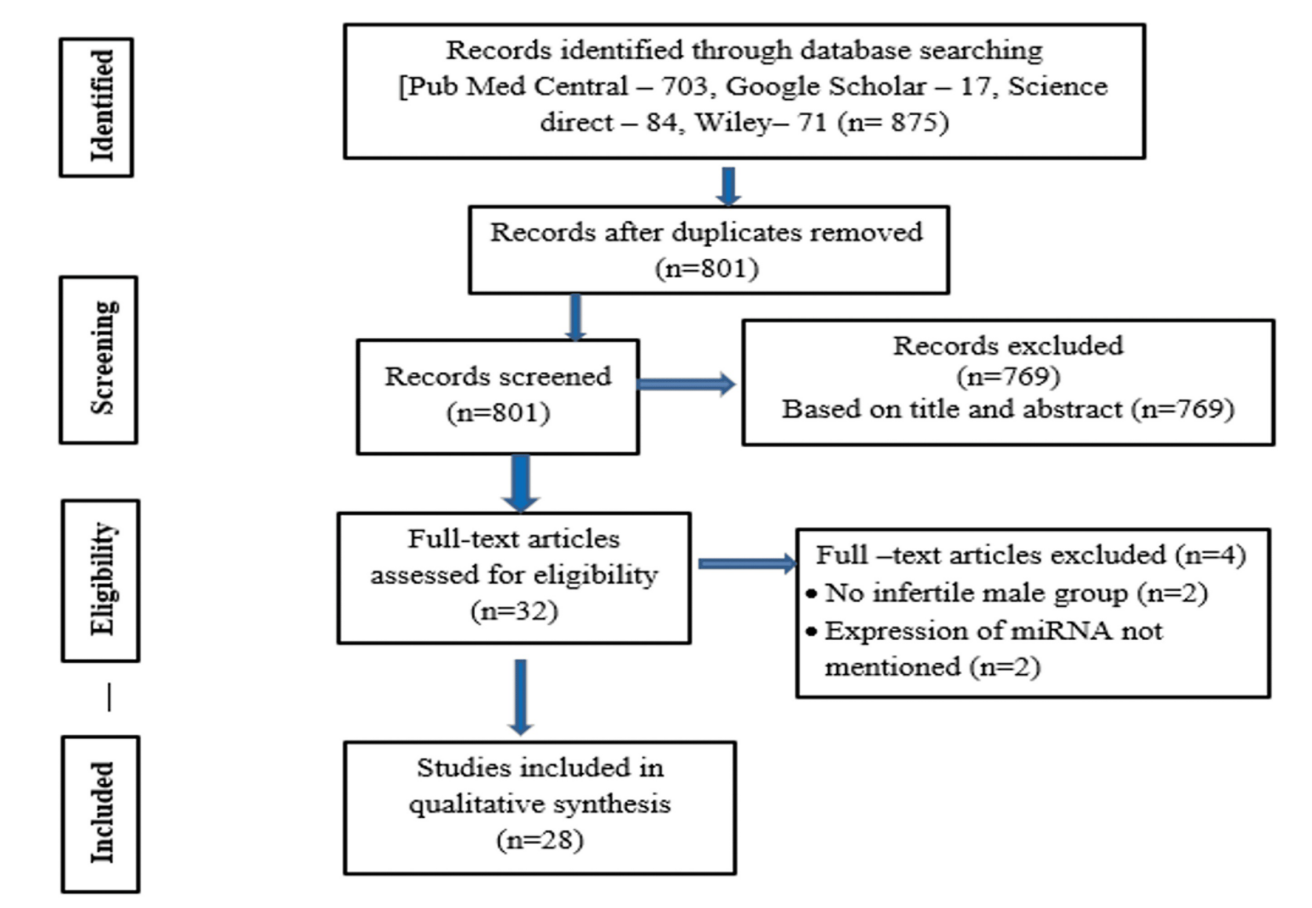
Study selection process following the Preferred Reporting Items for Systematic reviews and Meta-Analyses (PRISMA) flow chart

A total of 875 articles were retrieved from PubMed Central (703), ScienceDirect (84), Wiley (71), and Google Scholar (17). Seventy-four duplicate records were removed using reference manager software, and 769 title/abstract articles were excluded based on review criteria. Thirty-two studies underwent manual screening, and finally, 28 full-text articles that satisfied the inclusion criteria were encompassed in the qualitative synthesis, as illustrated in Figure 1. 

Characteristics of the Included Studies

Geography: Most studies were conducted in China (n = 14), Spain (n = 5), and Iran (n = 4), with fewer studies in Egypt and Canada.

Study design:Studies were predominantly case-control (n = 26); two studies did not specify design [20,21].

Participants: A total of 1,699 were infertile men; 1,239 were classified by sperm abnormality (asthenozoospermia: 459; azoospermia: 252; oligozoospermia: 228; others: varicocele, vasectomy, unexplained, unclassified). Fertile controls were 759.

Age: The mean age of infertile men was 27.5 ± 4.8 to 37 ± 7 years, while that of fertile men was 26 ± 3.3 to 36 ± 5 years.

Baseline sperm parameters: For asthenozoospermia, the parameters were reduced motility [22-26], decreased morphology [21-23], and reduced density [25]. For oligoasthenozoospermia, the parameters were low count and motility [20,21,27]. For asthenoteratozoospermia and OAT, it was abnormal motility/morphology [28,29]. DNA fragmentation was linked to reduced motility [20]. A summary of the characteristics of the included studies is presented in Table 1. 

**Table 1:** Characteristics of the included studies

First author [Ref]	Year	Country	Study design	Total sample size	Infertile men	Fertile men
Harchegani AB [9]	2018	Iran	Case-control	18	8	10
Abhari [15]	2014	Iran	Cross-sectional, case-control	86	43	43
Abu-Halima M [16]	2019	Germany	Case-control	66	33	33
Mostafa [17]	2016	Egypt	Case-control	220	125	95
Zhao K [20]	2015	China	Case-control	94	94	0
Salas-Huetos [21]	2015	Spain	Case-control	30	20	10
Zhou QZ [22]	2019	China	Case-control	60	30	30
Abu-Halima M [23]	2013	Germany	Case-control	27	18	9
Qing X [24]	2017	China	Case-control	44 (screening); 94 (validation)	22; 47	22; 47
Heidary Z [25]	2019	Iran	Case-control	74	39	35
Heidary [26]	2020	Iran	Case-control	85	45	40
Zhou JH [27]	2015	China	Case-control	50	48	42
Abu-Halima M [28]	2016	Germany	Case-control	24	12	12
Zhou JH [29]	2017	China	Retrospective case-control	40	20	20
Rahbar [30]	2020	Iran	Case-control	70	55	15
Carreau S [31]	2019	Spain	Case-control	23	14	9
Munoz [32]	2015	Spain	Case-control	16	9	7
Tian [33]	2017	China	Case-control	180	144	36
Ji Z [34]	2014	China	Case-control	20	10	10
Cui [35]	2015	China	Retrospective observational, case-control	202	162	40
Zhou R [36]	2015	China	Case-control	100	50	50
Zhou R [37]	2018	China	Case-control	100	50	50
Wang C [38]	2011	China	Case-control	254	186	68
Wu W [39]	2012	China	Case-control	240	192	48
Wu W [40]	2013	China	Case-control	40	20	20
Barceló M [41]	2018	Spain	Case and control prospective	36	27	9
Hu L [42]	2014	China	Case-control	7	3	4
Belleannee C [43]	2013	Canada	Case-control	11	8	3

Risk of Bias

**Figure 2:**
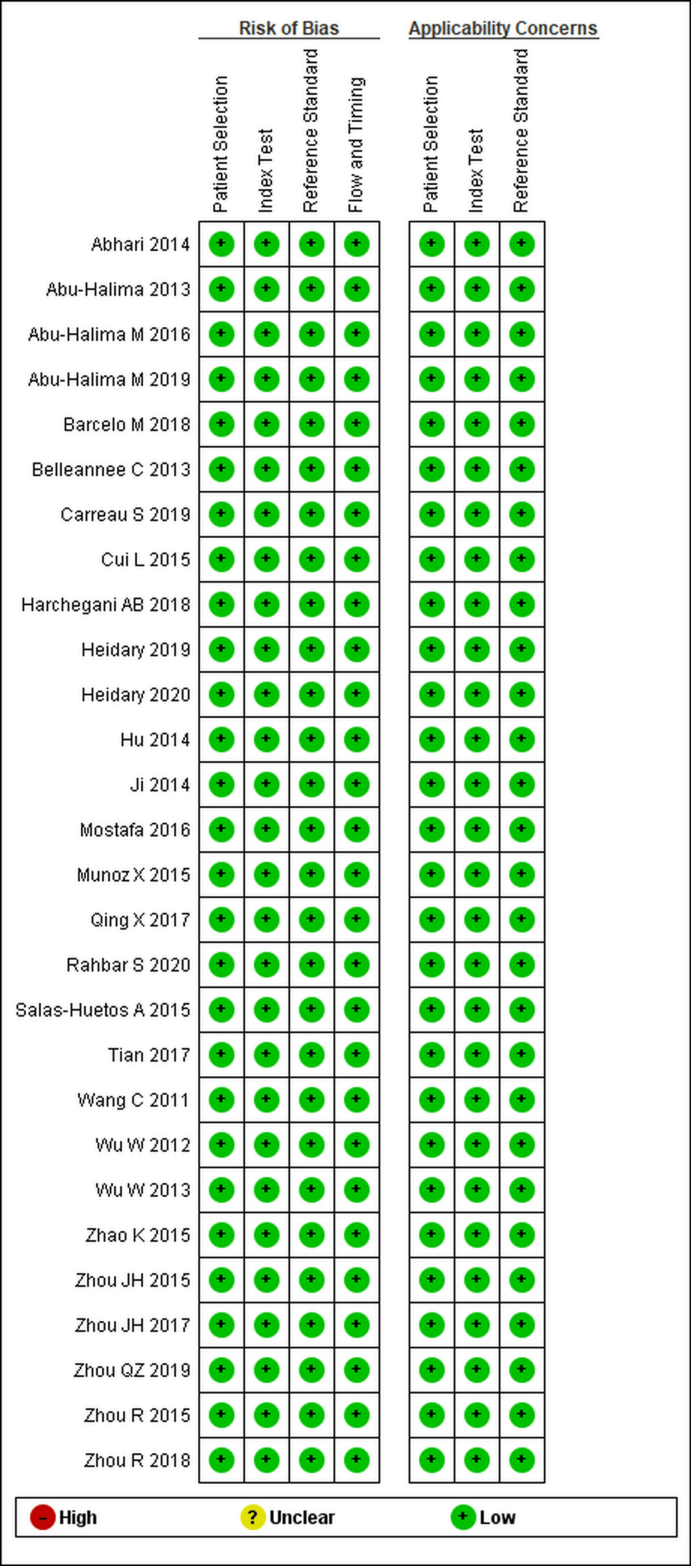
Risk of bias and applicability concerns of the included studies assessed using the QUADAS-2 tool Studies evaluated (in order shown in figure; first author, year): Abhari 2014 [15]; Abu-Halima 2013 [23]; Abu-Halima M 2016 [28]; Abu-Halima M 2019 [16]; Barceló M 2018 [41]; Belleannée C 2013 [43]; Carreau S 2019 [31]; Cui 2015 [35]; Harchegani AB 2018 [9]; Heidary Z 2019 [25]; Heidary 2020 [26]; Hu 2014 [42]; Ji 2014 [34]; Mostafa 2016 [17]; Muñoz X 2015 [32]; Qing X 2017 [35]; Rahbar S 2020 [30]; Salas-Huetos A 2015 [21]; Tian 2017 [33]; Wang C 2011 [38]; Wu W 2012 [39]; Wu W 2013 [40]; Zhao K 2015 [20]; Zhou JH 2015 [27]; Zhou JH 2017 [29]; Zhou QZ 2019 [22]; Zhou R 2015 [36]; Zhou R 2018 [37]. Legend symbols: green = low risk/concern; yellow = unclear; red = high risk This figure was created by the authors using Review Manager (Rev Man) version 5.4 software, developed by the Cochrane Collaboration. No copyrighted material was used.

All included studies were judged low RoB across the QUADAS-2 domains, namely, patient selection, index test, reference standard, and flow/timing, with low applicability concerns. The summary of the RoB is illustrated in Figure 2. 

Differential microRNA Expression Levels in Fertile and Infertile Men

Studies have shown that specific miRNAs are expressed differentially in infertile men as compared to fertile controls.

Spermatozoa: Oligozoospermia has increased miR-21, miR-22 [15]; decreased miR-10a/b, miR-16, miR-34b/c, miR-122, miR-135a/b, miR-888, miR-891a [21,27,29]. Asthenozoospermia has increased miR-888-3p, miR-141, miR-200a, miR-27b [21,24,30,31]; decreased miR-525-3p, miR-4485-3p, miR-122, miR-34b [20,21,26]. Oligoasthenozoospermia has increased miR-141, miR-200a, miR-23a/b-3p [16,21]; decreased miR-34b/c, miR-10a/b, miR-135a/b, miR-888, miR-891a [29]. Oligoasthenoteratozoospermia has increased miR-27a in asthenoteratozoospermia [27]; increased miR-15b; decreased miR-122, miR-383 [28]. Azoospermia has decreased miR-34b/c, miR-10a/b, miR-135a/b, miR-888, miR-891a [29]. Varicocele has decreased miR-15a [33]; decreased miR-122, miR-181a, miR-34c-5p in OAT [17]. Idiopathic infertility has decreased miR-34b/c [34]. 

Seminal plasma: Asthenozoospermia has decreased miR-101-3p, let-7b-5p, miR-891b, miR-892a/b, miR-888, miR-890; increased sp-miR-151a-5p, miR-34c-5p, miR-122, miR-146b-5p, miR-181a, miR-374b, miR-509-5p, miR-513a-5p [23,34-37]. Oligoasthenospermia has decreased miR-30b, miR-20a, miR-148a, miR-26b, miR-15a; increased miR-765, miR-1299, miR-1275 [26]. Azoospermia has decreased miR-34c-5p, miR-122, miR-146b-5p, miR-181a, miR-374b, miR-509-5p, miR-513a-5p [37]. Non-obstructive azoospermia has increased miR-19b, let-7a, miR-141, miR-429, miR-7-1-3p [38,39]; miR-19b most pronounced. Obstructive azoospermia has nine miRNAs increased and 42 decreased [40]. Sperm DNA fragmentation has increased miR-15b; decreased miR-424, miR-29c [20]. Post-vasectomy has decreased miR-514a-3p, miR-892b, miR-888, miR-34c-5p; increased miR-26b normalized after reversal [41,42]. 

Correlations With Sperm Abnormalities

Sperm count: miR-21/22 decreased in oligozoospermia [15]; miR-34b, miR-122 increased [29]; miR-122-5p increased in azoospermia [40].

Oligoasthenozoospermia: miR-23a/b-3p negatively correlated [16]. Oligoasthenoteratozoospermia: miR-122, miR-181a, miR-34c-5p increased [17]; miR-383 increased [28].

Asthenozoospermia: Positive correlation with miR-525-3p, miR-891b, miR-892a/b, miR-888, miR-890; negative correlation with miR-629-3p, miR-27b [20,21,23,25].

Morphology: miR-21/22 decreased in oligozoospermia [15]; miR-27b decreased, miR-525-3p increased in asthenozoospermia [21,25]; miR-122, miR-181a, miR-34c-5p increased in OAT [17]; miR-383 increased [28].

Sperm pH: miR-21/22 decreased [15].

Correlations With Genes/Gene Products

miR-22 positively correlated with estrogen (r = +0.665). miR-21/22 negatively correlated with ERβ (r = -0.834, -0.820) [15]. miR-15a decreased HSPA1B [33]. miR-122, miR-181a, and miR-34c-5p positively correlated with antioxidants and negatively with oxidative markers [17]. miR-23a/b-3p inversely correlated with PFKFB4, HMMR, SPATA6, TEX15 [16]. miR-27a/b negatively correlated with CRISP2; miR-525-3p negatively correlated with SEMG1 [21,25,27]. miR-151a-5p decreased ATP/Cytb [34]. let-7b-5p decreased glycolysis via AURKB [31]. miR-424 mediated DNA damage [20]. miR-184 and miR-383 correlated with CYCLIN D1 [28]. 

Discussion

**Figure 3:**
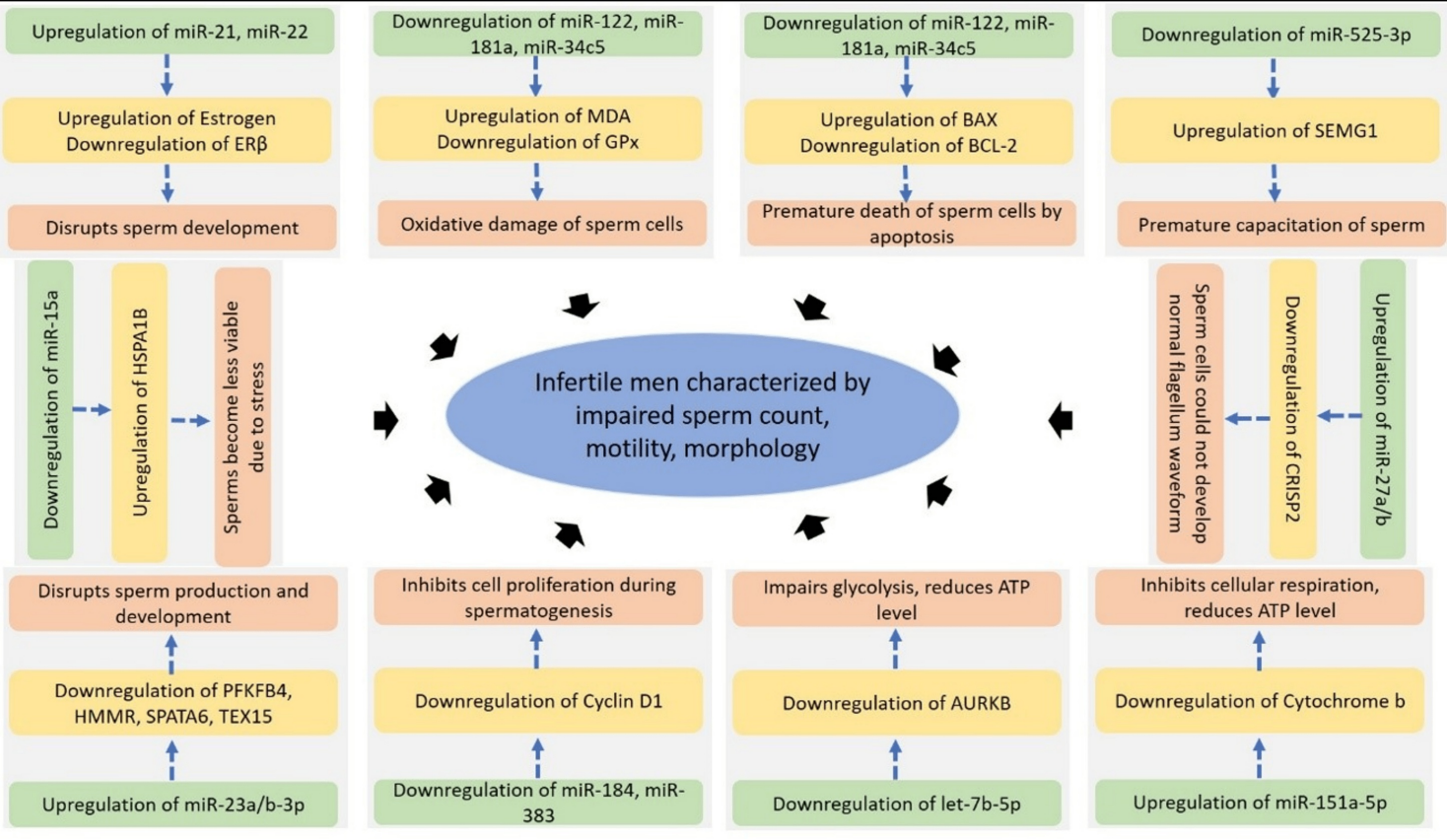
A visual representation of the role of distinct miRNAs on male infertility through regulating target genes/gene products This figure was created by the authors using Microsoft PowerPoint (Microsoft 365). No copyrighted material was used.

The present systematic review highlights the role of miRNAs in the regulation of male fertility through their associations with semen parameters such as sperm concentration, motility, morphology, and gene products. Differential expression of miRNAs and their expression is strongly correlated with impaired semen quality of infertile men compared to fertile controls, suggesting their potential utility as diagnostic biomarkers and therapeutic targets in male infertility (Figure 3).

Infertile men with low sperm count have higher miR-21/22 in spermatozoa, interfering with estrogen receptor signaling [16]. ERs are critical in spermatogenesis, sperm capacitation, and fertilization [44-46]. Oligoasthenoteratozoospermia shows reduced sperm count, motility, and morphology due to downregulation of miR-122, miR-181a, and miR-34c5, affecting BCL2-mediated apoptosis [17,46,47]. Higher miR-23a/b-3p levels correlate with reduced count, motility, and morphology via SPATA6 and TEX15 downregulation [17, 27].

miRNAs also regulate motility: CRISP2 is key in sperm motility and acrosome reaction; miR-27a suppresses CRISP2, reducing motility in asthenozoospermia [27,29,48]. Mitochondrial ATP production is essential for motility; defects impair sperm function [25,49]. 

Specific miRNAs act as positive or negative regulators, indicating that infertility arises from an imbalance in a miRNA network. miRNA evaluation in spermatozoa and seminal plasma offers advantages over traditional semen analysis due to stability and predictive capacity. The stability of miRNAs in biological fluids further enhances their popularity as non-invasive biomarkers for diagnosing male infertility in clinical settings [50]. 

Limitations

Most included studies employed case-control designs, limiting the ability to infer causality. High-throughput sequencing and microarray studies were excluded unless validated by qPCR, potentially overlooking novel miRNAs. Furthermore, most studies focused on a limited set of miRNAs, whereas male infertility likely involves complex interactions among multiple miRNA networks and other regulatory molecules. Sample sizes were often small, and ethnic or geographic differences may affect generalizability. There are also chances of language bias.

## Conclusions

This systematic literature review demonstrates that specific miRNAs are differentially expressed in infertile men compared to fertile controls, with some being upregulated and others downregulated. These altered expression patterns are significantly associated with genes and gene products involved in sperm formation, maturation, and development. Such evidence highlights the regulatory role of these non-coding RNAs in sperm characteristics and supports their potential as non-invasive diagnostic biomarkers for the evaluation of male infertility.
